# Who is donating to political parties in Queensland, Australia? An analysis of political donations from the food industry

**DOI:** 10.1017/S1368980023000435

**Published:** 2023-07

**Authors:** Cherie Russell, Nurul Amanina Binte Hussain, Katherine Sievert, Katherine Cullerton

**Affiliations:** 1 School of Public Health, The University of Queensland, 266 Herston Rd, Brisbane, Herston, QLD 4006, Australia; 2 School of Exercise and Nutrition Sciences, Deakin University, Geelong, Australia

**Keywords:** Political donation, Food industry, Corporate political activities, Political economy, Nutrition policy, Commercial determinants of health

## Abstract

**Objective:**

Australia’s dominant food system encourages the overconsumption of foods detrimental for human and planetary health. Despite this, Australia has limited policies to reduce the burden of disease and protect the environment. Political donations from the food industry may contribute to policy inertia on this issue. We aimed to explore the extent of political donations made by the food industry in Queensland and investigate the timing of public health nutrition policies in relation to these donations.

**Design:**

We collected publicly declared political donations data in Queensland, Australia, as it has the most transparent donation records. Policy data were sourced from the Australian National and Queensland State Parliaments, and consultations from the Australian and New Zealand Ministerial Forum on Food Regulation.

**Setting:**

Queensland, Australia.

**Participants:**

Not applicable.

**Results:**

The Liberal National Party (LNP) received 68 % of all donations, with most immediately preceding the 2017 and 2020 state elections. The Australian Labor Party, despite forming government for the time period under study, received only 17 % of total donations. Most donations were given by the meat industry, followed by the sugar industry. Few policies exist to protect and improve human and planetary health, with limited associations with political donations for most industries except sugar.

**Conclusions:**

Industry preference for the LNP, particularly as most donations coincided with election periods, may be due to the party’s emphasis on minimal state involvement in economic and social affairs. The relationship between industry donations and policies is not clear, partly due to the limited number of policies implemented overall.

Despite providing large quantities of safe food and economic benefits, Australia’s dominant industrialised food system creates a consumptogenic food environment that promotes poor dietary patterns^(1)^. Typical Australian diets are characterised by low intakes of fruits, vegetables and whole grains, and excessive consumption of red/processed meat and ultra-processed foods^(2,3)^. Consequently, Australians have high rates of diet-related non-communicable diseases accounting for an estimated 87 % of deaths annually^(2)^. This non-communicable disease ‘epidemic’ has significant economic and social ramifications, with costs of approximately $120 billion (AUD) annually due to a loss of productivity and well-being^(4)^. Poor dietary patterns are not only a leading risk factor for Australia’s burden of disease^(5)^ but also contribute to various forms of environmental degradation, including greenhouse gas emissions, resource depletion and biodiversity loss^(6,7)^.

To attenuate these issues, comprehensive, multi-sectoral policy actions are needed to reduce the availability and affordability of food that is detrimental for human and planetary health. However, to date, Australia has limited policy actions regarding food to reduce non-communicable disease rates and protect the environment^(8)^. Most policy actions that have been implemented are voluntary rather than mandatory, including food reformulation targets and a front-of-pack labelling system, or have involved consumer education^(8,9)^. Unsurprisingly, the food and beverage corporations that may be impacted by such policy have resisted and opposed more severe and mandatory policy proposals^(8,10)^.

Strategies used by parts of the private sector to promote their interests and the consumption of harmful products have contributed to Australia’s political inertia regarding public health nutrition issues^(8,11)^. Such strategies include conducting public relations campaigns for corporate social responsibility initiatives; utilising lobbyists and media communications; creating strategic public–private alliances; pursuing litigation; creating front groups and providing political donations^(11)^. The provision of political donations is a strategy that is particularly well-suited to the commercial sector in that they are often well-resourced in comparison to public-health focused organisations^(12)^. Previous research suggests that political donations from harmful commodity industries, including fossil fuels, tobacco and gambling, influence policymaking by creating favourable market conditions for those industries and potentially eroding the democratic process^(13–16)^. It is important to note that here we refer to segments of the food industry that financially benefit from food and beverages that, in currently consumed quantities, are detrimental to human health, rather than the food industry as a whole.

In Australia, substantial donations have been made to political parties from harmful commodity industries, ranging from $1 million donated by the tobacco industry (2005–2015) to $7 million from the alcohol industry^(12–14)^. Though political donations from the tobacco industry are no longer accepted by The Australian Liberal or Labor Parties, others including The National Party and Independents parliamentarians do still accept such donations. However, the prevalence and impact of political donations from the food industry are under investigated. Documents analysed in 2013 and 2014 demonstrate donations of $55 000 from Coca-Cola to Australian political parties, while a major Australian supermarket chain donated more than $35 000^(17)^. However, a systematic examination of food industry donations has not been conducted in Australia, nor has the association between these political donations, political events and relevant policy outcomes been established.

One reason for this literature gap is that the process of reporting political donations within Australia is far from fully transparent. At the federal level, only donations to political parties that exceed $13 800 are required to be formally recorded. These donations are then made public on an annual basis, including the donor’s name, recipient and donation amount^(18)^. Similar reporting requirements occur in most states. Comparatively, the state of Queensland requires donation disclosure for gifts above $1000 and is the only state with real-time reporting (donations are publicly disclosed within 7 d of receipt)^(18)^. To date, no studies have used this dataset to investigate donations from the food industry to political parties, including who they are donating to and the potential implications this may have on policy outcomes. Therefore, we aim to explore the extent of political donations made by the food industry in Queensland and investigate the timing of public health nutrition policies in relation to these donations.

## Method

To analyse the value of political donations made by the food industry in Queensland and their potential relationship with public health nutrition policy, we collected three types of data: (i) publicly declared political donations in Queensland; (ii) legislation tabled in the Australian National Parliament, the Queensland State Parliament and (iii) consultations from the Australian and New Zealand Ministerial Forum on Food Regulation (Forum). The Forum is responsible for setting the food policy framework for Australia and consists of health and agriculture ministers from the states and territories, and the Australian and New Zealand governments^(19)^.

### Data collection

#### Political donations in Queensland

We chose to analyse political donations (henceforth referred to as donations) made in Queensland as it is the only Australian state with real-time reporting. Data were collected from the electronic disclosure system on the Electoral Commission of Queensland website^(20)^. We downloaded all donations published from 1^st^ January 2016 to 30^th^ November 2021 as a Microsoft Excel (V.2108) spreadsheet. Available information for each donation included the donor’s name, the recipient, the date of the donation and the donation value. Due to a change in reporting practices, additional information including the donor electorate and donor address was available for entries prior to 2019 only. In the Australian context, an electorate is defined as a specific geographical area represented by one member of parliament.

To determine the identity and relevance of each donor, we performed a comprehensive web search using the donor’s name, address and electorate (where available). Key terms (including ‘*Queensland, farm, sugar, cattle, meat, salt, food and drink*’), determined iteratively by scoping relevant literature and Government Hansard transcripts, were searched along with donor information to increase the number of donors we could identify. Donations were included if we were able to determine the donor’s identity, and if the donor pertained to any of the three groups of the food sector hierarchy sourced from the Australian Institute of Health and Welfare^(21)^ (AIHW) (Table 1).

**Table 1:** The Australian Institute of Health and Welfare food sector hierarchy^(21)^

Tier	Description	Example
Primary	Agriculture and fishing	Cattle station, sugarcane farm
Secondary	Processors and manufacturers	Meat processor, sugarcane miller
Tertiary	Retailers	Supermarket, hospitality venue

To avoid misrepresentation and overestimation of results, we excluded entries where there was any uncertainty about the identity and nature of the donor. Donations from unions were also excluded, as these organisations predominantly advocate for employment-related matters, such as wages and working conditions, which falls beyond the scope of this study^(22)^. For our analysis, we have classified each donation by political party, rather than individual candidates. For example, if a Liberal party candidate received a donation, we categorised this as a donation for the Liberal party. Only donations to four key political parties in Queensland were included in the analysis, as these parties are deemed to have the highest level of influence in policymaking in Queensland due to their large proportion of seats in parliament^(23)^. We did not include the Australian Greens in our analysis due to their policy on not accepting alcohol donations^(24)^ and a lack of donations from the food industry to this party. A description of each party ideology, expanded upon from Russell *et al*.^(25)^, is shown in Table 2. For the timeframe under study, the Australian Labor Party (ALP) was the governing party in Queensland.

**Table 2:** Queensland political party ideologies

Political party	Ideology/ies
Liberal National Party (LNP) (merger between the Liberal and National party)	Liberal ideology: emphasis on minimal state involvement. Any government intervention is to help people help themselves^(29)^ Neoliberal ideology: free market economics (that the economy works best when left alone by the government); unregulated market capitalism delivers efficiency, growth and widespread prosperity^(44)^ Agrarianism/Country mindedness: emphasises supporting the rights and enhancing the status of farmers; supports financial and social incentives for farmers^(45–46)^.
Australian Labor Party (ALP)	Social democracy: aims to ‘humanise’ capitalism by striving for a balance between market economy and state intervention^(29)^; economic and social interventions can rectify the shortcomings of capitalism; the state is the custodian of public interest and social change can and should be brought about peacefully and constitutionally^(47)^; support for government-funded welfare and taxation schemes
Katter’s Australian Party (KAP)	Agrarianism/Country mindedness: emphasises supporting the rights and enhancing the status of farmers; supports financial and social incentives for farmers^(45–46)^ Economic nationalism: favours state interventions over market mechanisms, including domestic control of the economy^(48)^ Right-wing populism: juxtaposes social classes, aims to defend a national culture and identity against perceived attacks by outsiders^(49)^.
Pauline Hanson’s One Nation (One Nation)	Ultra-nationalism: xenophobia, supports authoritarian politics^(50)^ Right-wing populism: juxtaposes social classes, aims to defend a national culture and identity against perceived attacks by outsiders^(49)^

#### Policy mapping of Queensland public health nutrition issues

To determine potential government policy actions that may have been influenced by donations, we conducted a purposive search of the Bills and Acts recorded on the Queensland State and Commonwealth Government’s Parliamentary website. State election dates were identified via the Queensland Parliament website^(26)^. This was supplemented with food regulation consultations from the Forum, sourced from their website. All consultations, bills and acts initiated between 2016 and 2021 were included in the analysis if they related to public health nutrition. Here, we define public health nutrition as a population-based focus on health promotion, food and nutrition systems, wellness maintenance and primary prevention^(27)^.

### Data analysis

After screening the donations data, donors were coded by sector type based on the AIHW groups (Table 1). If an organisation covered multiple sectors, the donor was coded according to their principal role, determined through grey literature searching and team discussion. Donations were also coded by their industry, including meat (consisting of cattle, pork, sheep and poultry), sugar, fruit and vegetables, nuts, alcohol, seafood, non-alcoholic beverages (juice, soft drinks, coffee, tea, water), peak industry organisations representing the packaged food industry, hospitality (cafes, restaurants and takeaway food services), food retailers (supermarkets, wholesalers), grains, dairy and eggs. A randomised 10 % sample was coded independently by a second researcher to reduce bias, with an inter-reliability of >90 %. Team discussions occurred to resolve discrepancies.

Details from relevant bills and acts tabled in the Queensland State and Australian National parliaments and in Forum consultations were extracted and categorised in a Microsoft Excel spreadsheet. Categories included the name of the proposed legislation/consultation, the date initiated or assented to, a description of content, the party that tabled it (if applicable) and its status. Relying on the expertise of the authors in food and nutrition policy, food systems and corporate political activity, we undertook a double coding of all policies to determine which group, if any, where most likely to benefit from the policies. Any conflicts were resolved through team discussion. We then organised the data chronologically to synthesise the policies with the political donations data from the seven industries who gave the most money between 2016 and 2021. Descriptive statistics and graphical outputs to represent the data were generated using Microsoft Excel.

## Results

### Political donations in Queensland

**Fig. 1:**
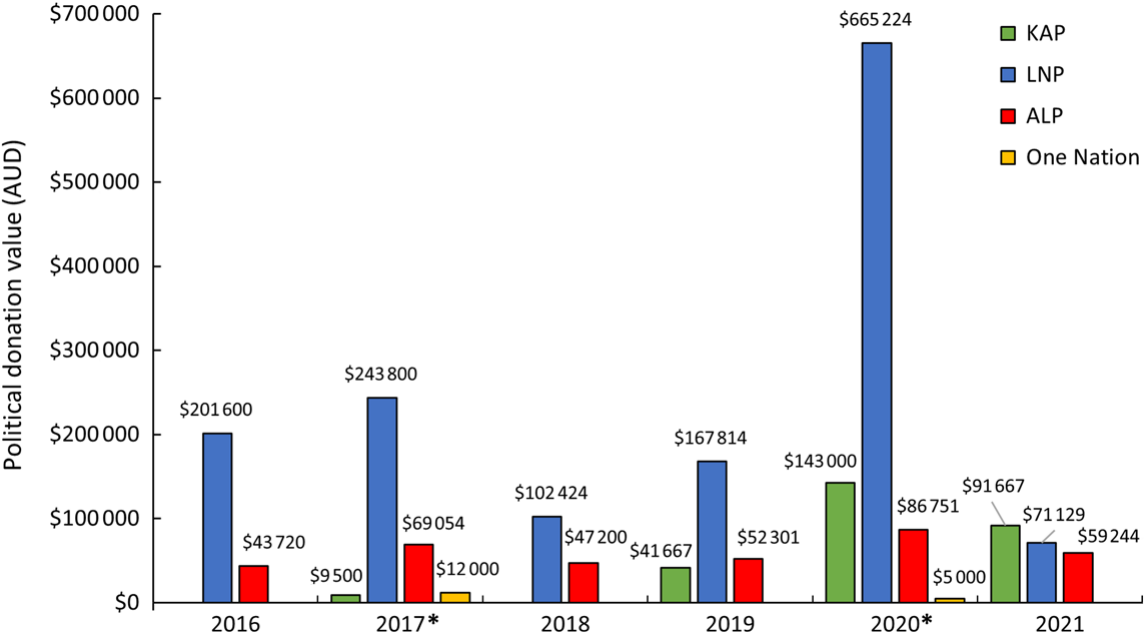
* – year of state election. Political donations (AUD) from the food industry to the four main political parties in Queensland, 2016–2021. KAP, Katter’s Australian Party; LNP, Liberal National Party; ALP, Australian Labor Party; One Nation, Pauline Hanson’s One Nation Party

Of the 12 661 publicly declared political donations recorded between January 2016 and November 2021 in Queensland, 526 were made by 212 unique representatives of the food industry, totalling $2 113 093. These donations were declared under the names of large local organisations, trans-national corporations, small private businesses and individuals. Donations ranged in value from $40 to $60 000. The Liberal National Party (LNP) received 68 % of all donations ($1 451 991), while the ALP received 17 % ($358 270), Katter’s Australian Party (KAP) received 14 % ($285 833) and One Nation received <1 % ($17 000) (Fig. 1). Most parties had the highest net donations in 2020, except One Nation, which had a peak in donations in 2017.

Peaks in money donated to the LNP, KAP and One Nation occurred immediately preceding state elections (Fig. 1, see online Supplemental 1). There were also notable peaks in donations to the LNP between May and July in 2016–2020, corresponding with Budget Week and Estimate Committee Hearing Week (see online Supplemental 1). These two events are important to capture as Queensland’s Members of Parliament discuss how they will allocate the federal budget given to the State^(28)^, examine their Ministerial Portfolio statements and discuss how they will spend the allocated budget within their department^(28)^.

**Fig. 2:**
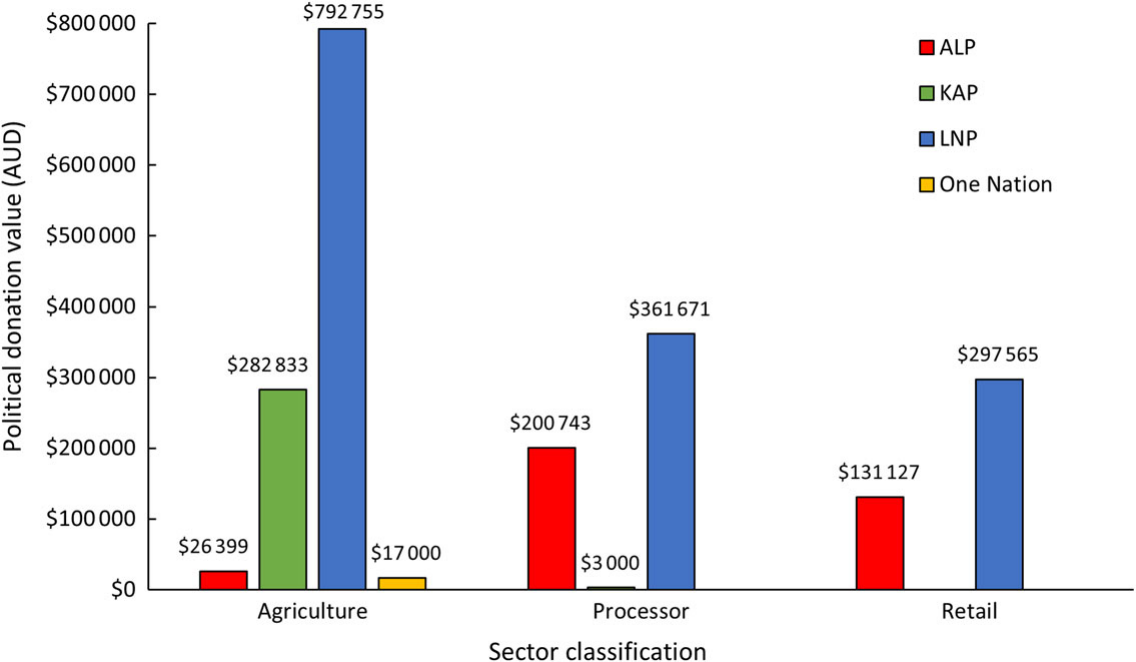
Political donations to major parties in Queensland by sector, 2016–2021. KAP, Katter’s Australian Party; LNP, Liberal National Party; ALP, Australian Labor Party; One Nation, Pauline Hanson’s One Nation Party

A significant proportion of the total donations were made by representatives from the agriculture sector (53 %), followed by processors (27 %) and the retail sector (20 %) (Fig. 2). The LNP received the highest net donations from all three sectors, with the highest amount ($792 755; 55 % of donations received by the LNP) being donated by the agriculture sector. All donations to One Nation, and almost all to KAP, were also from the agriculture sector. Comparatively, most donations to the ALP were from processors ($200 743, 56 % of donations received by the ALP).

**Fig. 3:**
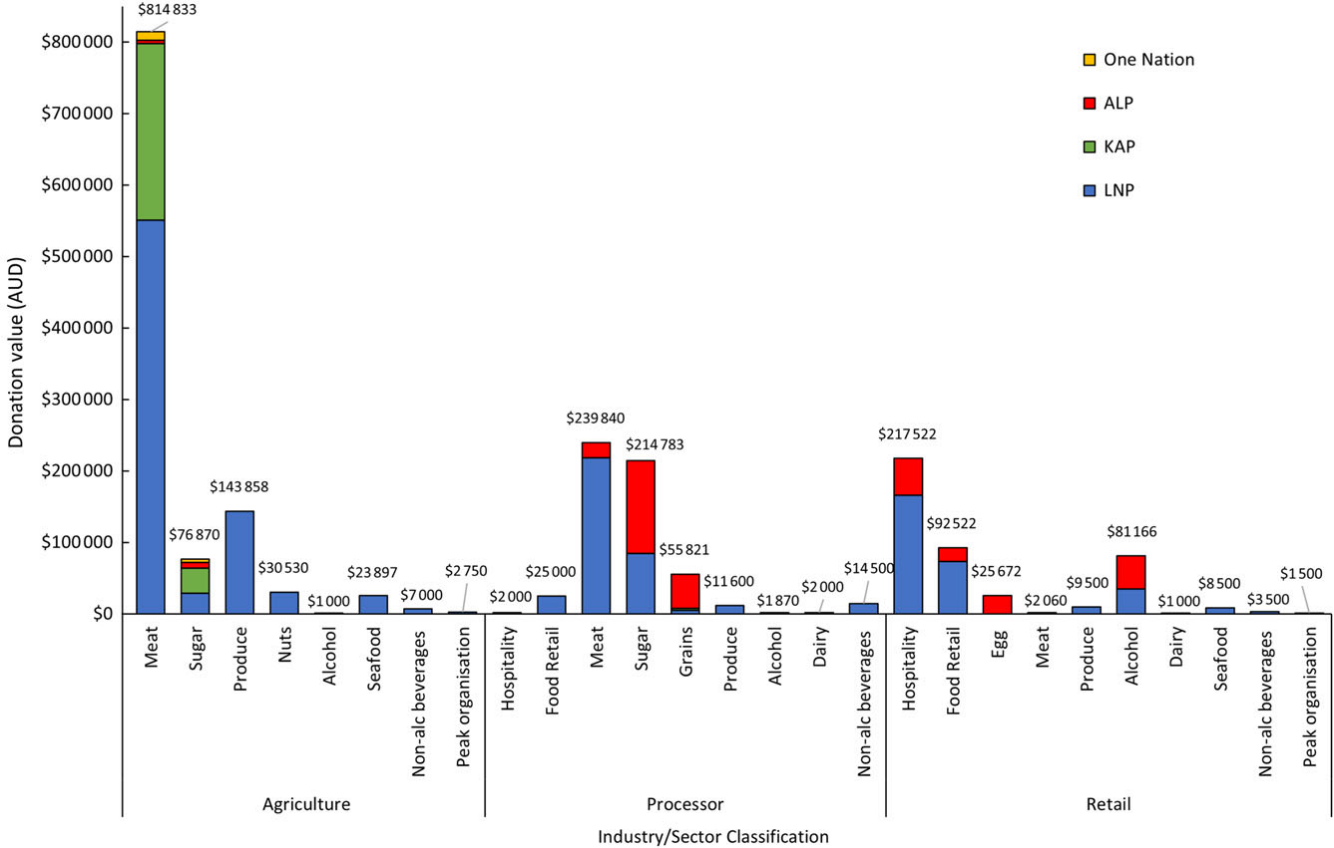
Political donations to major parties in Queensland by sector and industry, 2016–2021. KAP, Katter’s Australian Party; LNP, Liberal National Party; ALP, Australian Labor Party; One Nation, Pauline Hanson’s One Nation Party

Most donations were given by the meat industry ($1 056 733, 50 %), followed by the sugar industry ($291 653, 14 %) (Fig. 3, see online Supplemental 2). These industries donated to all parties, although the LNP, KAP and One Nation mostly received donations from the meat industry ($770 600, 53 % of LNP donations; $247 333, 87 % of KAP donations and $12 000, 70 % of One Nation donations respectively). Most money donated to the ALP was from the sugar industry ($137 362, 38 % of ALP donations). The produce, seafood, nut, non-alcoholic beverage and dairy industry only donated to the LNP. Comparatively, the egg industry only donated to the ALP.

The highest aggregated donation overall ($233 333) was made to KAP by a relative of the party head who co-owns a cattle farm in Queensland. This donor also had the highest frequency of donations in the time period under study. The second largest donor (‘Gulf Coast’, another cattle farm) had an aggregated donation of $171 800, given almost exclusively to the LNP. Over half (*n* 127) of the food industry donors donated only once between 2016 and 2021, seventy-two donated ten or less times and seven donated more than 10 times. Nineteen donated to multiple political parties, though mostly to the LNP and ALP. Only one donor (the Pioneers Cane Grower Organisation) donated to all political parties.

### Policy mapping of Queensland public health nutrition issues

**Fig. 4:**
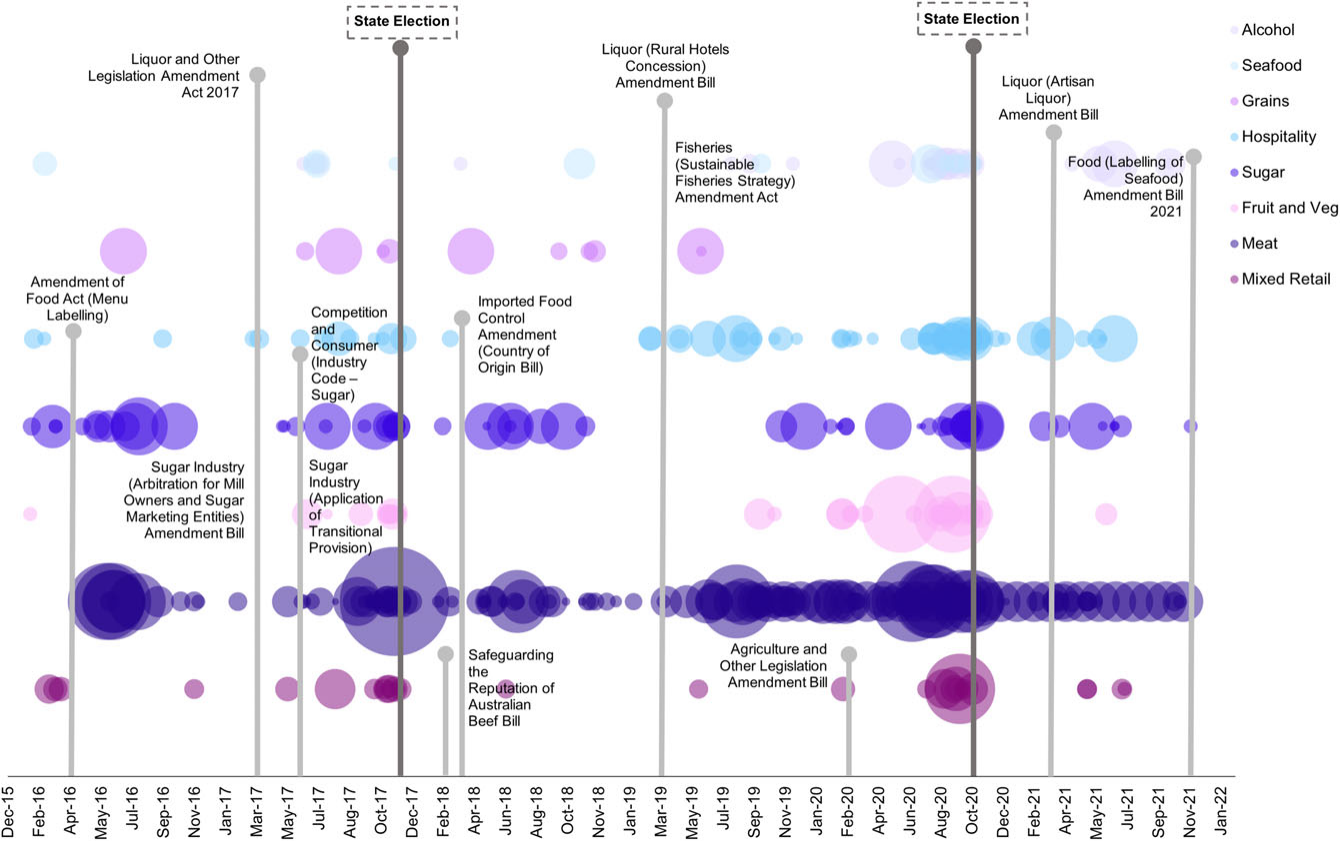
Value of political donations for each food industry gifted between 2016 and 2021 mapped against relevant policies assent/withdrawal date. Larger circles represent more money donated

Details of each act, bill and consultation related to food, including the name, description, who introduced the legislation (if applicable), the outcome and the date of this outcome, are shown in Table 3. These results are synthesised with political donations from the seven industries with the highest value of donations data in a timeline below, including relevant Queensland policies and elections (Fig. 4). Peaks in donations were mostly observed preceding State elections, with little correlation between increased industry donations and relevant policies for most industries. However, concentrations in donations increased in tandem with proposed legislation and consultation relevant to sugar. This included increased donations preceding the *Sugar Industry (Arbitration for Mill Owners and Sugar Marketing Entities) Amendment Bill* and the *Labelling of sugars on packaged foods and drinks consultation,* and immediately following the tabling of the *Competition and Consumer Sugar Industry Code Bill.*

**Table 3:** Legislation tabled in Queensland related to meat, sugar, fruit and vegetables, hospitality and food retail between 2016 and 2021

Bill/proposal name (year introduced)	Introduced by	Description	Outcome	Date of outcome	Stakeholder group most likely to benefit
Amendment of Food Act (Menu Labelling) (2015)	ALP	Amended the *Food Act 2006* to provide for the display of nutritional information for food. The menu labelling scheme in *the Food Act* requires certain food businesses that sell ready-to-eat food to display on their menus, both in-store and where distributed electronically and in print, the average kilojoule content of each standard food or drink item that they sell, and an average energy intake statement for adults	Passed	March 2016	Population Health; Food Industry
Sugar Industry (Application of Transitional Provision) Amendment Bill (2017)	One Nation	An Act to amend the *Sugar Industry Act 1999* to extend the period within which particular supply contracts are not required to include particular terms	Withdrawn	June 2017	Food Industry
Sugar Industry (Arbitration for Mill Owners and Sugar Marketing Entities) Amendment Bill (2017)	LNP	An Act to amend *the Sugar Industry Act 1999* to ensure sugar mill owners and sugar marketing entities undertake negotiations in a fair, timely and business-like manner to finalise ‘on supply’ agreements	Failed	March 2017	Food Industry
Liquor and Other Legislation Amendment Act (2017)	ALP	Amends the *Liquor Act 1992*, addresses the findings of the Tackling Alcohol-Fuelled Violence Legislation Amendment Act 2016 (TAFV Act) interim evaluation report including repealing lock outs for licensed venues	Assented	March 2017	
Competition and Consumer (Industry Code – Sugar) (Pre 2017)	LNP	Regulates the conduct of growers, mill owners and marketers (of grower economic interest sugar) in relation to contracts or agreements for the supply of cane or the on-supply of sugar. This includes establishing a process for pre-contractual arbitration where the parties fail to agree to terms of contracts or agreements.	Tabled	May 2017	Food Industry
Energy labelling of alcoholic beverages (2016)	The Forum	Consultations regarding the energy labelling of alcoholic beverages	Ongoing	n/a	Population Health
Fresh produce food safety risk management (2017)	The Forum	Developed with stakeholders to facilitate stakeholder discussions regarding food safety management and the fresh produce supply chain	Complete	September 2017	Population Health; Food Industry
Safeguarding the reputation of Australian Beef Bill (2017)	Independent	Penalises Australian cattle exporters that do not take reasonable steps to ensure that Australian cattle that is slaughtered, or processed after slaughter in a foreign country, is not marketed as Australian beef	Removed from Notice Paper	February 2018	
Imported Food Control Amendment (Country of Origin Bill) (2017)	LNP	Amends the *Imported Food Control Act 1992* to incorporate the Country-of-Origin Food Labelling Information Standard 2016 by reference	Assented	March 2018	
Review of fast-food menu labelling schemes (n/a)	The Forum	Consultations regarding the effectiveness of the fast-food menu labelling schemes since the release of the endorsed National Principles for Introducing Point-of-Sale Nutrition Information in Standard Food Outlets	Ongoing	n/a	Population Health; Food Industry
Labelling of sugars on packaged foods and drinks (2018)	The Forum	Consultation on a range of policy options on the labelling of sugars on packaged foods and drinks	Ongoing	n/a	Population Health; Food industry
Regulation impact statement for pregnancy warning labels on packaged alcoholic beverages (pre-2017)	The Forum	Assessed stakeholder views on mandatory labelling standard for pregnancy warning labels on packaged alcoholic beverages	Completed	October 2018	Population Health
Liquor (rural hotels concession) amendment bill (2018)	KAP	Changes the existing rigid liquor licencing framework to reflect the unique circumstances of licensed venues in very remote communities	Assented	March 2019	Food Industry
Fisheries (sustainable fisheries strategy) amendment act (2018)	ALP	Modernise the objectives of the *Fisheries Act 1994* and recognise the interests of key stakeholder groups; clarify the roles of the Minister responsible for fisheries and the chief executive in the management of the State’s fisheries; strengthen enforcement powers and penalties to address serious fisheries offences such as black-marketing	Assented	March 2019	Population Health
Agriculture and Other Legislation Amendment Bill (2019)	ALP	‘Omnibus’ Bill addresses a number of impediments, identified over the past several years, to the efficient and effective regulation of agriculture; animal management and welfare; forestry; and fisheries	Assented	February 2020	Population Health; Food Industry
Stakeholder engagement – implementation of recommendations from the health star rating system 5-year review report (2019)	The Forum	This consultation process sought advice on a number of implementation parameters, and develop an Implementation Plan for changes to the Health Star Rating system	Complete	October 2020	Population Health; Food industry
Liquor (Artisan Liquor) Amendment Bill (2020)	ALP	Amends the *Liquor Act 1992* to: create a new liquor license category for legitimate craft brewers and artisanal distillers; expand existing capabilities for selling artisanal products at promotional events and encourage the transition of existing licensees to the new artisan producer license category	Assented	March 2021	Food Industry
Policy guidance for menu labelling in Australia and New Zealand (2019)	The Forum	In this consultation, FRSC sought stakeholder views to inform the development of policy guidance and an effective policy framework for consistent menu labelling.	Under review	June 2021	Population Health; Food Industry
Food (labelling of seafood) amendment bill (2021)	KAP	Requires by law mandatory Country of Origin Labelling of seafood sold in the food service sector, through “dining outlets”, across Queensland	Introduced	November 2021	Population Health; Food Industry

## Discussion

In this study, we aimed to explore the extent of political donations made by the food industry in Queensland and investigate the timing of public health nutrition policies in relation to these donations. Our results suggest that the LNP is the preferred political party for the food industry in Queensland. Of Australia’s two primary parties, the LNP is more conservative and has a greater emphasis on minimal state involvement in economic affairs^(29)^. In addition to their conservative ideology, the National Party faction of the LNP was established to represent and protect farmers. The bias displayed by Australia’s food industry in favour of a conservative party could demonstrate a ‘conservative partisanship’ strategy^(30)^. This approach is typically adopted by industries facing hostile regulation, as conservative governments tend to advocate for the preservation of existing social and economic structures^(30)^. Furthermore, the inclusion of the National Party in the LNP may in part explain the high value of donations from the agriculture sector.

In contrast, a ‘pragmatic’ strategy of bipartisan hedging in political donations is more likely to be adopted by industries that are already regulated, or for whom regulation is ‘inevitable’^(30)^. Industries donate in a bipartisan manner to gain access to regulators regardless of who is in power^(30)^. For example, we found that the sugar industry, one of Queensland’s most lucrative crops, donated approximately equally to both the ALP and LNP. Globally, sugar is an increasingly politicised crop, with a growing focus on the impact of added sugars to the global burden of disease^(31)^. In the time period captured in this study, four bills and one consultation specifically relating to sugar were introduced. Interestingly, we saw a division between sugarcane growers and processors. Growers were more likely to follow the trend of agricultural organisations and individuals donating to the LNP, while processors were more likely to donate to the ALP. A potential reason for this disparity could include proposed regulation from the ALP to protect the Great Barrier Reef against environmental harms from sugar processing^(32)^. Although this pattern of donating was observed in our study, it was not as prevalent as the ‘conservative partisanship’ strategy.

Our policy mapping demonstrates that there were limited legislated policies or consultations in Queensland and Nationally between 2016 and 2021 to improve human and planetary health. Most of the legislation that was implemented related to issues of agricultural processes rather than decreasing the consumptogenic nature of the food supply or the environment. It was therefore difficult to ascertain whether political donations impacted the outcome of any specific bill or act tabled in the period under study. Though there was limited association between increased industry donations and relevant policies for most industries, donations from the sugar industry, the second biggest donator, coincided with three potential policies. However, none of these polices or consultations resulted in legislative action. It is not possible from our study to explain the influence that these donations had on policy outcomes, as there are limited data, though donations may have been a contributing factor in preventing the progression of policies and consultations that could negatively impact the sugar industry.

The limited implementation of evidence-based policy suggests that part of the apparent influence of the food industry in Queensland is their ability to keep public health issues off the political agenda. Instead, most donations, particularly those given to the LNP, were received immediately preceding the 2017 and 2020 state elections, with donations significantly lower in non-election years. This is comparable to research of Australian political donations overall^(33)^. The cost of running election campaigns in Australia is rising, including the advertising of a party’s political agenda to try and gain electoral support^(34,35)^. Increasing costs encourage a reliance on external funding, including via political donations^(13,34,35)^, which account for 30–50 % of political party income^(30,35)^.

Our findings mirror historical trends in donations from the tobacco and alcohol industries, which increase during federal election periods and preceding political discussions and decisions for regulatory reforms^(12,13,17)^. These donations may be provided with the hope that industry ideologies and commercial interests will be represented by their preferred political party^(13,30,34,35)^. Donations can influence the policymaking process by facilitating increased access to politicians and consequently, allowing representatives from the harmful commodities sector to develop long-term relationships with these decision makers^(12,15,34,36)^. Research suggests that industry representatives who donate multiple times within a year are more likely to have meetings or contact with politicians^(30,34)^. This gives commercial entities the opportunity to talk about industry-related issues or gain a ‘sympathetic hearing’, which may potentially influence a political outcome^(35,36)^. Meeting with big or frequent donors can be a way politicians express gratitude and secure donations for future elections^(13,35)^.

Most donations overall, and to the LNP, KAP and One Nation, were provided by the meat industry. The meat industry – particularly beef, sheep and goat meat – is one of Australia’s largest sources of economic revenue and is a major export commodity to the Asia-Pacific region, generating around $18 billion in revenue each year^(37)^. Consequently, the relationship between government and the meat industry has been largely interdependent and symbiotic. Policy interests have largely focused on maintaining production and consumption of meat, despite the growing environmental concerns associated with greenhouse gas emission outputs, water and land use and reduced agrobiodiversity^(38)^. Industry representative groups, like Meat and Livestock Australia, receive substantial levies from government, as well as agricultural subsidies of over $200 million/year^(39)^. At the time of writing, no major party at either Federal or State level has taken any health or environmental policy position in relation to the impact of meat production^(40)^. The targeting of LNP, KAP and One Nation is likely due to the majority seats held in rural electorates, where cattle farming and meat processing plants are run and employed by sizeable portions of these populations.

Other than sugar and meat, there was a lack of declared donations from harmful commodity industries, including alcohol and ultra-processed food corporations. Though our results demonstrate that such industries are not donating to political parties in the state of Queensland, future research should investigate their donations in other states and at the national level. One reason these industries may not be donating to political parties is their size and subsequent economic power. The food industry is one of the highest employing sectors in the state and contributes substantially to Queensland’s economy^(41)^. Politicians may hesitate to implement policies that could jeopardise these economic resources^(15)^. The prioritisation of economic interests in policymaking over public health and sustainability is well documented^(42)^. Furthermore, regulating the food industry may have limited social license as a voting issue, could lead to issues with trade agreements and goes against the continuing pressures to support personal choice and to promote personal responsibility^(42,43)^. Advocacy from public health organisations can encourage major parties to give up harmful industry funding, as evidenced by major political parties no longer accepting donations from the tobacco industry. These findings have important implications for policy regarding transparency and integrity in the future. We would encourage restrictions on the size of donations that can be made to political parties, as well as developing a standardised process for political donations transparency between states and at a national level. To expand upon the analysis presented in this paper, future research should consider the course of a bill over time, including suggested amendments; industry lobbying for each policy issue and if/how the content of a finalised bill differs from when it was initially tabled.

This is the first Australian study to measure political donations from the food industry in Queensland, and the first to map these donations against relevant legislation to assess potential relationships. Our study was limited by the analysis of only one state, rather than multiple state or national political donations. This choice was made due to the lack of real-time donation data and the quality of donations data nationally and in other states. Delayed reporting of political donations and poor limited data common is common in Australia^(33)^. Of particular concern is the Australian Electoral Commissions publication of political donations, which can have an 18-month gap between the date of a donation and the information release^(35)^. We acknowledge that many factors are involved in policymaking and political donations are only one aspect of this. Therefore, our findings regarding associations between donations and policy outcomes need to be considered in this complex policymaking environment. There may also be other policy that was not captured in our food-related search, particularly regarding the trade, agriculture and economic policies. Further, while Queensland donation data are generally considered high quality, it was difficult to determine the exact identity of some donors as limited detail was provided in the disclosure statements, leading to potential underestimations in our analysis.

## Conclusion

The LNP was the preferred political party for the food industry in Queensland. This is potentially because it is the more conservative of Queensland’s two major parties, with an emphasis on minimal state involvement in economic affairs and preservation of existing social and economic structures. Donations were not regularly correlated with policies or consultations for most industries, rather they mostly preceded state elections. However, we found limited policy implemented overall to reduce food industry harms on human and planetary health, with most legislation related to issues of agricultural processes rather than decreasing the consumptogenic nature of the food supply or environment. Most donations were from the meat and sugar industries. Both industries may face regulatory intervention in the future given the relationship between these commodities and health and environmental outcomes.

## Supplementary material

For supplementary material accompanying this paper visit https://doi.org/10.1017/S1368980023000435. click here to view supplementary material
