# Supplementary material for: Who is donating to political parties in Queensland, Australia? An analysis of political donations from the food industry
Source: Public Health Nutr. 2023 Mar 1;26(7):1501–12. doi: 10.1017/S1368980023000435 (PMC10346088; doi:10.1017/S1368980023000435)

**Supplement 1.** Political donations, in Australian Dollars (AUD), made by the Food Industry in different months within the 6 year period (solid line). ⯁ = Budget week. KAP – Katter’s Australian Party. LNP – Liberal National Party; ALP – Australian Labor Party; One Nation – Pauline Hanson’s One Nation Party


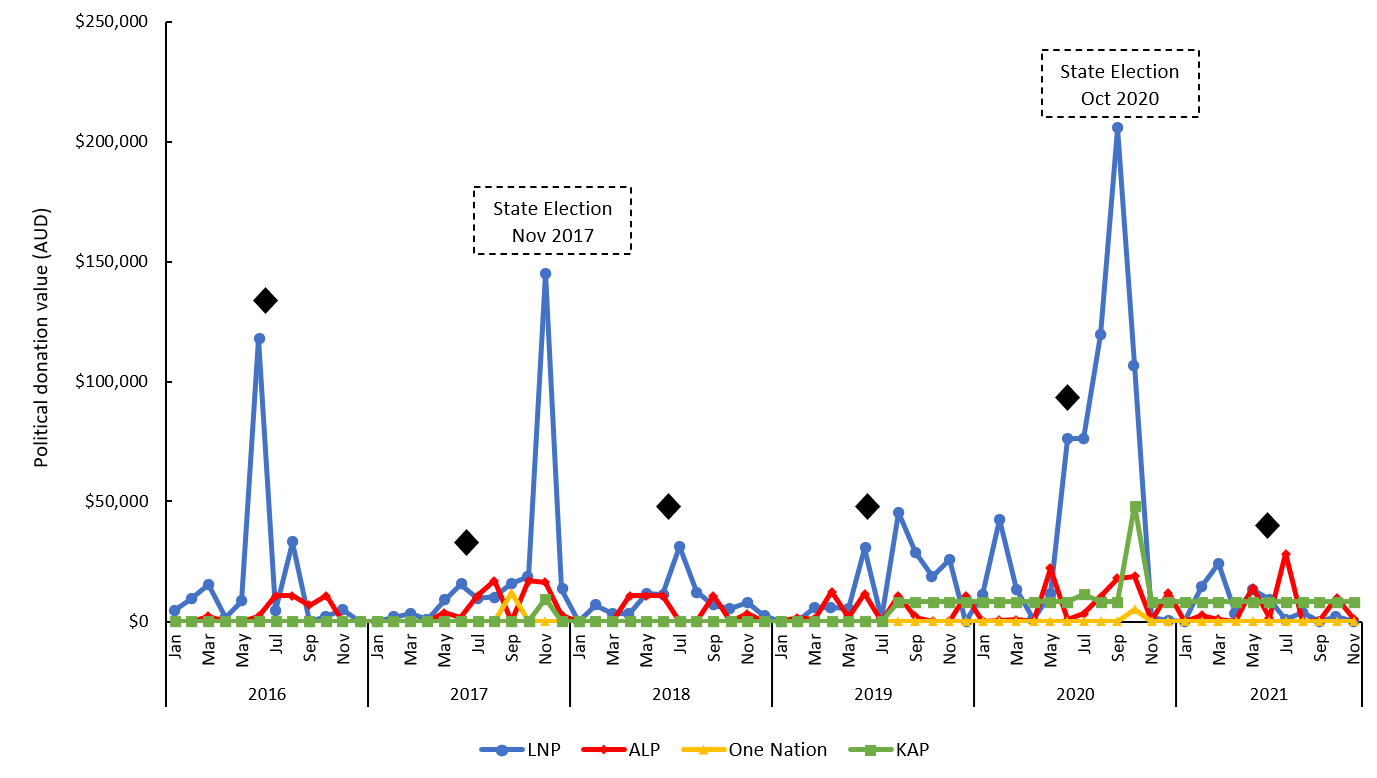

Supplement: Supplementary file 1 [file S1368980023000435sup.zip › S1368980023000435sup001.docx]
