# Supplementary material for: Who is donating to political parties in Queensland, Australia? An analysis of political donations from the food industry
Source: Public Health Nutr. 2023 Mar 1;26(7):1501–12. doi: 10.1017/S1368980023000435 (PMC10346088; doi:10.1017/S1368980023000435)
Supplement: Supplementary file 1 [file S1368980023000435sup.zip › S1368980023000435sup002.docx]

**Supplement 2:** Political donations to major parties in Queensland by sector and industry, 2016 – 2021. KAP – Katter’s Australian Party. LNP – Liberal National Party; ALP – Australian Labor Party; One Nation – Pauline Hanson’s One Nation Party

|  | **Donations received by each political party** | | | | |  |
| --- | --- | --- | --- | --- | --- | --- |
| **Sector/Industry** | **LNP donations** | **KAP donations** | **ALP donations** | **One Nation donations** | **Total donations** | |
| **Agriculture** | **$792,755** | **$282,833** | **$12,150** | **$17,000** | **$1,104,738** | |
| Alcohol | $1,000 |  |  |  | $1,000 | |
| Hospitality | $2,000 |  |  |  | $2,000 | |
| Livestock | $550,850 | $247,333 | $4,650 | $12,000 | $814,833 | |
| Peak organisation | $2,750 |  |  |  | $2,750 | |
| Sugar | $28,870 | $35,500 | $7,500 | $5,000 | $76,870 | |
| Seafood | $23,897 |  |  |  | $23,897 | |
| Produce | $143,858 |  |  |  | $143,858 | |
| Non-alc beverages | $7,000 |  |  |  | $7,000 | |
| Nuts | $30,530 |  |  |  | $30,530 | |
| Seafood | $2,000 |  |  |  | $2,000 | |
| **Processor** | **$361,671** | **$3,000** | **$200,743** |  | **$565,414** | |
| Alcohol |  |  | $1,870 |  | $1,870 | |
| Grains | $4,900 | $3,000 | $47,921 |  | $55,821 | |
| Livestock | $218,750 |  | $21,090 |  | $239,840 | |
| Mixed Retail | $25,000 |  |  |  | $25,000 | |
| Sugar | $84,921 |  | $129,862 |  | $214,783 | |
| Produce | $11,600 |  |  |  | $11,600 | |
| Non-alc beverages | $14,500 |  |  |  | $14,500 | |
| Dairy | $2,000 |  |  |  | $2,000 | |
| **Retail** | **$297,565** |  | **$145,376** |  | **$442,941** | |
| Alcohol | $34,829 |  | $46,337 |  | $81,166 | |
| Egg |  |  | $25,672 |  | $25,672 | |
| Hospitality | $166,116 |  | $51,406 |  | $217,522 | |
| Livestock | $1,000 |  | $1,060 |  | $2,060 | |
| Mixed Retail | $73,120 |  | $19,402 |  | $92,522 | |
| Peak organisation |  |  | $1,500 |  | $1,500 | |
| Seafood | $8,500 |  |  |  | $8,500 | |
| Produce | $9,500 |  |  |  | $9,500 | |
| Non-alc beverages | $3,500 |  |  |  | $3,500 | |
| Dairy | $1,000 |  |  |  | $1,000 | |
| **Total received** | **$1,451,991** | **$285,833** | **$358,269** | **$17,000** | **$2,113,093** | |
